# Modification of Aluminum Hydroxide by Ball Milling: A Feasible Method to Obtain High-Efficiency Flame Retardants for Production of High-Performance EVA Composites

**DOI:** 10.3390/ma18050984

**Published:** 2025-02-24

**Authors:** Man Yang, Bihe Yuan

**Affiliations:** 1 School of Environmental Science and Engineering, Hubei Polytechnic University, Huangshi 435003, China; 204076@hbpu.edu.cn; 2 School of Safety Science and Emergency Management, Wuhan University of Technology, Wuhan 430070, China

**Keywords:** flame retardant, aluminum hydroxide, ball milling modification, ethylene–vinyl acetate copolymer

## Abstract

Aluminum hydroxide (ATH) is an environmentally friendly flame retardant widely employed in polymers. However, the high loading of ATH, due to its limited efficiency, potentially compromises other properties, including mechanical properties. This work explores a feasible ball milling strategy for high-efficiency ATH-based flame retardants (PPA-ATH and PPOA-ATH), fabricated by employing phenylphosphinic acid (PPA) and phenylphosphonic acid (PPOA) as surface modifiers and water as the processing solvent. The characterization study of PPA-ATH and PPOA-ATH demonstrates that ball milling effectively reduces their particle size, enhances their specific surface area, and improves their dispersibility within the ethylene-vinyl acetate (EVA) matrix. PPOA-ATH exhibited superior capabilities in enhancing the thermal stability and flame retardancy of EVA composites compared to PPA-ATH. The incorporation of PPOA-ATH resulted in the retarding in the temperature at 50% mass loss by 21 °C and an increase in the char residue of 34.5% at 700 °C. Furthermore, PPOA incorporation led to reductions of 81.0% in the peak heat release rate, 48.1% in the total heat release, 73.7% in the peak smoke production rate, and 41.2% in the total smoke production compared to neat EVA. This green modification strategy successfully addresses the application limitations of ATH, providing a feasible and environmentally friendly method for high-efficiency ATH-based flame retardant fabrication.

## 1. Introduction

Aluminum hydroxide (ATH) serves as a crucial inorganic flame retardant, widely employed in various sectors, such as plastics, rubber, and textiles, due to its cost-effectiveness and environmentally friendly characteristics [1]. Under high-temperature conditions, ATH decomposes into aluminum oxide (Al_2_O_3_) and water vapor. The released water vapor dilutes the flame and lowers the combustion temperature, thus providing a cooling effect [2]. The aluminum oxide generated from this decomposition forms a dense protective layer on the material’s surface, inhibiting further heat and oxygen transfer, which effectively suppresses flame propagation [3].

The advantages of ATH as a flame retardant are primarily ascribed to its environmental friendliness, non-toxicity, and efficient smoke suppression [4]. Nonetheless, its flame retardancy is generally inferior to that of many organic flame retardants, necessitating a high loading exceeding 50 wt% to achieve the desired flame retardant performance [5]. Furthermore, excessive incorporation of ATH leads to poor compatibility with polymer materials owing to its polarity, potentially compromising other material properties, including mechanical strength [6,7]. Recent studies have demonstrated that modification represents an effective strategy to enhance flame retardant efficiency and improve the compatibility of inorganic flame retardants within the matrix [8,9].

Ball milling is a facile approach to modification that utilizes the mechanical energy generated by the impacts and friction of milling media within a ball mill [10,11]. It has gained widespread adoption in flame retardant fabrication due to its advantages of easy processing, low cost, and large-scale production [12]. Furthermore, ball milling modification facilitates significant changes, including surface area enhancement, particle size reduction, and dispersion effect improvement [13]. As reported in Xu’s work [14], ball milling modification of attapulgite effectively increases its specific surface area by 218% and improves its dispersion effects. Moreover, during the ball milling process, modifiers can more readily react with the surfaces of nanoparticles, resulting in the introduction of specific functional groups and enhancement in their properties [10]. For example, Wang et al. [15] prepared MB-BNNS by grafting highly efficient flame retardant elements onto boron nitride surfaces via ball milling, resulting in a uniform dispersion within the epoxy resin and enhanced flame retardancy and thermal conductivity. He et al. [16] fabricated a hybrid flame retardant (MA) by introducing amino groups onto red phosphorus through ball milling, followed by encapsulation to prepare MA microcapsules (MRP/MA). The synthesized flame retardant system exhibited a uniform morphology, low moisture absorption, and minimal toxic gas release. As a simple and effective mechanical synthesis method, ball milling allows for precise control over the microstructural and surface characteristics of materials at the nanoscale. However, the application of ball milling for ATH modification remains relatively unexplored.

This study aimed to employ a simple, feasible, and high-yield ball milling modification method for the rapid surface modification of ATH. This approach involved the grafting of phenylphosphinic acid (PPA) and phenylphosphonic acid (PPOA) onto the surface of ATH. The modified ATH was subsequently be incorporated into ethylene–vinyl acetate (EVA) copolymer. It was anticipated that the surface modification would enhance the dispersion of ATH, as well as its flame retardant properties. Mechanochemical approaches, such as ball milling, offer promising avenues for enhancing the performance of ATH, ultimately facilitating its broader application in flame retardant formulations.

## 2. Materials and Methods

### 2.1. Materials

EVA (260, vinyl acetate content of 28 wt%) was purchased from DuPont de Nemours, Inc. (Wilmington, DE, USA). ATH, phenylphosphinic acid (PPA), and phenylphosphonic acid (PPOA) were acquired from China National Pharmaceutical Group Chemical Reagent Co., Ltd. (Shanghai, China).

### 2.2. Ball Milling Modification of ATH

**Figure 1:**
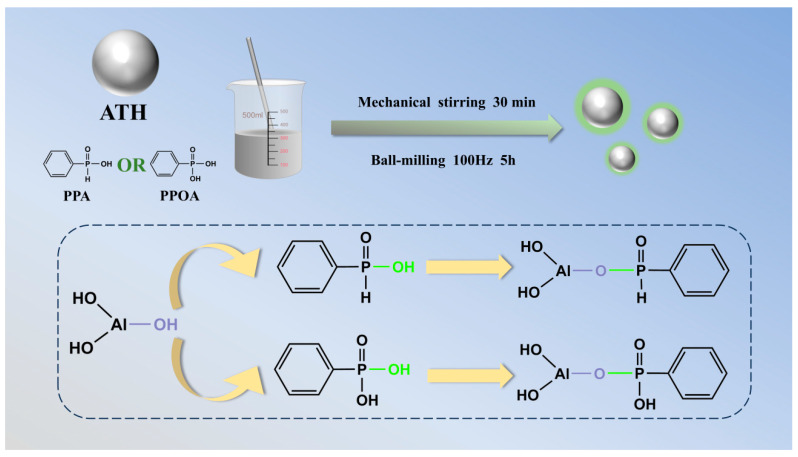
Schematic of PPA-ATH and PPOA-ATH preparation.

As shown in Figure 1, the surface modification of ATH was performed using a one-step ball milling method. Specifically, 100 g of ATH and 10 g of PPA were mixed with 50 mL of ultra-pure water under mechanical stirring for 30 min. The ball mill with 900 g of milling beads operated at a rotation speed of 25 Hz for a duration of 5 h. Upon completion of the milling process, the mixture was thoroughly washed three times with deionized water, followed by vacuum filtration. This final product (phenylphosphinic acid-treated ATH, PPA-ATH) was then dried in an oven at 80 °C for 24 h. The modification procedure for phenylphosphonic acid-treated ATH (PPOA-ATH) followed the same steps as described above. Notably, the entire modification process utilized water as the solvent, avoiding the environmental pollution associated with conventional organic solvent-based modification methods.

### 2.3. Preparation of EVA Composites

To remove the adsorbed moisture, EVA and the flame retardant were dried in a vacuum oven at 80 °C for 10 h prior to blending. The composite was then melt-blended with 50 wt% of the flame retardant using a QE-70A internal mixer produced by Wuhan Qien Technology Development Co., Ltd. (Wuhan, China). The blending process was conducted at a temperature of 150 °C for 7 min, with a rotor speed of 60 rpm. Subsequently, the resulting mixture was hot-pressed at 150 °C and 10 MPa pressure using a YF-8017 plate vulcanizer from Yangzhou Yuanfeng Experimental Machinery Factory (Yangzhou, China) for 5 min to obtain standard samples.

### 2.4. Characterization

The Fourier-transform infrared spectroscopy (FTIR) spectra of ATH, the modified ATH, and the char residues of the EVA composites were collected via the KBr pellet method using a Nicolet iS20 spectrometer from Thermo Scientific (Waltham, MA, USA). The test was carried out with a spectral resolution of 0.019 cm^−1^, a scanning range of 4000 to 400 cm^−1^, and a rapid-scan measurement rate of 1 scan per second.

X-ray photoelectron spectroscopy (XPS) analysis of PPA-ATH and PPOA-ATH was performed using a Thermo Fisher Scientific electron spectrometer (Waltham, MA, USA).

X-ray diffraction (XRD) patterns of ATH, PPA-ATH, and PPOA-ATH were obtained by an X-ray diffractometer (D8 Advance, Bruker AXS, Walzbach, Germany) with Cu Kα radiation (λ = 0.15418 nm) in a 2θ range from 10° to 90°.

The microstructure of the samples was observed using a TESCAN MIRA LMS scanning electron microscope (SEM, Kohoutovice, Czechia) at an accelerating voltage of 20.0 kV. Among them, ATH, PPA-ATH, PPOA-ATH, and the freeze-fractured surface of EVA and its composites were gold-sprayed before SEM observation.

Energy dispersive X-ray spectroscopy (EDS, SZEISS GeminiSEM 300, Oberkochen, Germany) of the samples was conducted at a beam energy of 15 keV. The SEM observation was carried out under the conditions of U = 5 kV and X (magnification) = 4000.

The particle size distributions of ATH and the modified ATH were measured using a BT-9300SE laser particle size analyzer from Dandong Bettersize Instrument Co., Ltd. (Dandong, China).

The hydrophilicity of ATH and the modified ATH was evaluated using a JY-PHb water contact angle (WCA) goniometer from Shanghai Bangyi Precision Instruments Co., Ltd. (Shanghai, China).

Thermal gravimetric analysis (TGA) of the samples (approximately 6.0 mg) were analyzed with an STA 6000 simultaneous thermal analyzer from PerkinElmer (Waltham, MA, USA). The test was carried out under a nitrogen atmosphere with a gas flow rate of 19.8 mL/min and heated at a rate of 20 °C/min over a temperature range of 50 to 800 °C.

The specific surface area (S_BET_) of ATH, PPA-ATH, and PPOA-ATH was determined by nitrogen adsorption–desorption isotherm measurements on a Brunauer–Emmett–Teller (BET, ASAP2460, Micromeritics Instrument Corp, Norcross, GA, USA) analyzer.

The tensile properties of EVA and its composites (1 mm thick) were assessed using a WDW-50M microcomputer-controlled electronic universal testing machine (Jinan, China), following the ASTM D638 standard. Dumbbell-shaped specimens were tested with a stretch rate of 20 mm/min.

The UL-94 vertical burning rating of the EVA composites was determined using an M607 horizontal–vertical burning apparatus from Qingdao Shanfang Instrument Co., Ltd. (Qingdao, China), in accordance with the ASTM D3801 standards. The dimensions of the standard specimens were 127 × 12.7 × 3.0 mm^3^.

The combustion behavior and thermal release parameters of EVA and its composites were investigated using a MOTIS cone calorimeter (Motis Fire Technology Co., Ltd., Suzhou, China), referring to the ISO 5660-1 standard. The samples, with dimensions of 100 × 100 × 3.0 mm^3^, were ignited with exposure to a vertical heat flux of 35 kW/m^2^.

## 3. Results and Discussion

### 3.1. Characterization of PPA-ATH and PPOA-ATH

**Figure 2:**
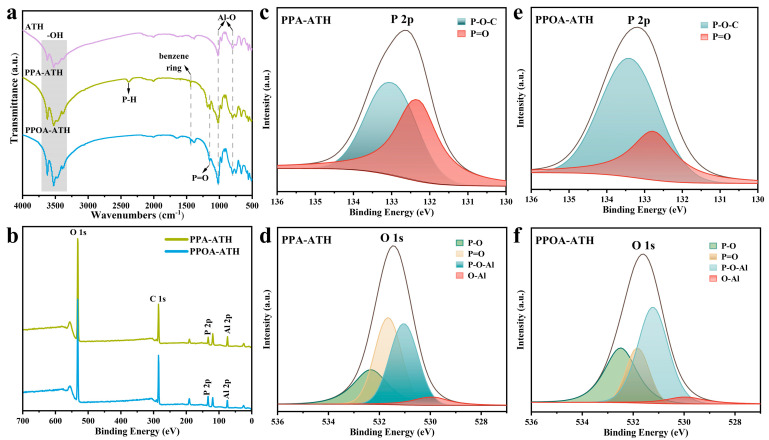
(**a**) FTIR spectra of ATH, PPA-ATH, and PPOA-ATH. XPS survey spectra: (**b**) full-scan spectra of PPA-ATH and PPOA-ATH; (**c**) P 2p and (**d**) O 1s spectra of PPA-ATH; (**e**) P 2p and (**f**) O 1s spectra of PPOA-ATH.

The chemical structures of PPA-ATH and PPOA-ATH were confirmed through FTIR and XPS analysis. As displayed in Figure 2a, the FTIR spectrum of ATH reveals characteristic peaks corresponding to -OH stretching vibration around 3500 cm^−1^ and Al-O groups at 1020 and 795 cm^−1^, indicating its typical structure [17]. After ball milling modification, new peaks appeared at 2390 cm^−1^ (P-H stretching vibration), 1440 cm^−1^ (benzene ring), and 1150 cm^−1^ (P = O stretching vibration), indicating the successful incorporation of the flame retardants [18,19]. XPS analysis was further conducted to investigate the elemental compositions of modified AHP. As shown in Figure 2b–f, the XPS spectra of PPA-ATH and PPOA-ATH exhibit similar peaks in the P 2p and O 1s spectrum. Specifically, the fitting of the P 2p pattern reveals binding energy at 132.5 eV and 133.2 eV, corresponding to P = O and P-O-C, respectively [20]. Moreover, the O 1s spectrum exhibits four significant peaks at 530.1 eV, 531.2 eV, 531.8 eV, and 532.4 eV, indicating the presence of O-Al, P-O-Al, O = P, and P-O, respectively [21,22]. These results indicate the successful grafting of PPA/PPOA onto ATH.

**Figure 3:**
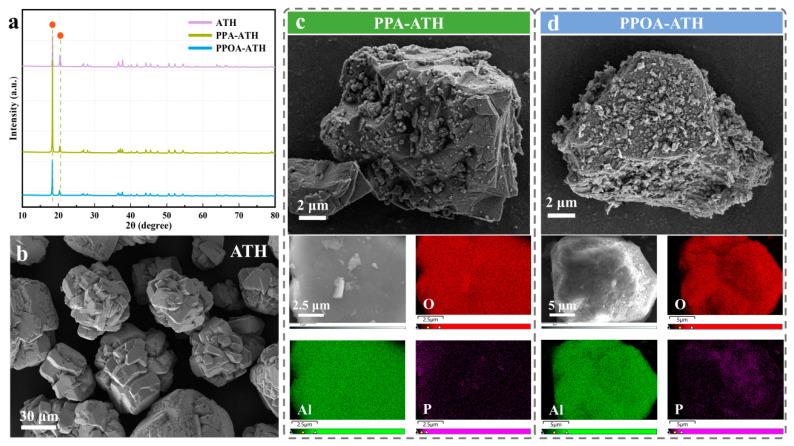
(**a**) XRD patterns of ATH, PPA-ATH, and PPOA-ATH. (**b**) SEM images of ATH. SEM, EDS, and elemental mapping images of (**c**) PPA-ATH and (**d**) PPOA-ATH.

The XRD patterns of ATH, PPA-ATH and PPOA ATH are illustrated in Figure 3a to study their crystalline structures. The diffraction peaks of pristine ATH at 18.36° and 20.36° correspond to its standard crystalline structure [23]. Following modification, the XRD patterns of both PPA-ATH and PPOA-ATH maintained these peaks without significant alteration, indicating that the modification preserved the crystal structure of ATH.

To further investigate the surface morphology of ATH, PPA-ATH, and PPOA-ATH, SEM, EDS, and mapping imaging were conducted. As depicted in Figure 3b, ATH exhibited a well-dispersed structure with smooth surfaces. After modification, both PPA-ATH and PPOA-ATH exhibited reduced particle dimensions and distinct surface roughening, indicative of successful modification. The EDS mapping image of the P characteristic element confirms the presence of PPA/PPOA, further demonstrating the successful grafting of PPA and PPOA onto the ATH surface.

**Figure 4:**
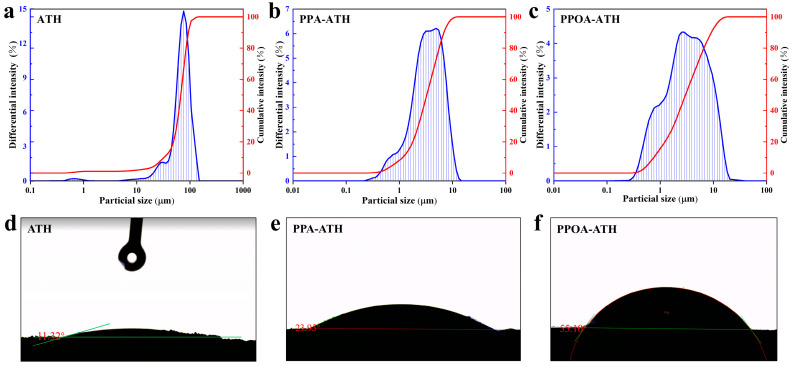
(**a**–**c**) Particle size distribution and (**d**–**f**) WCA results of ATH, PPA-ATH, and PPOA-ATH.

The particle size distribution curves of ATH, PPA-ATH, and PPOA-ATH are illustrated in Figure 4a–c. The particles of ATH primarily range in size from 10 to 110 μm, with an average particle size of 71.01 μm. After ball milling modification, the particle sizes of PPA-ATH and PPOA-ATH significantly decreased, both falling within the range of 0.5 to 10 μm and reaching an average particle size of 3.448 and 3.143 μm, respectively. These results are consistent with those obtained from the SEM images, indicating the effective effect of ball milling modification in reducing the particle size. The reduced particle size suggests better dispersion within the composite matrix, which is further discussed in the following section.

WCA tests were employed to evaluate the hydrophobicity of flame retardants. As shown in Figure 4d–f, the ATH was hydrophilic, with a WCA of only 11.32°. Following modification, the hydrophobicity of the modified ATH increased, with a WCA of 23.95° for PPA-ATH and 55.10° for PPOA-ATH. This improvement in hydrophobicity can be attributed to the incorporation of lipophilic benzene rings from PPA and PPOA [24].

**Figure 5:**
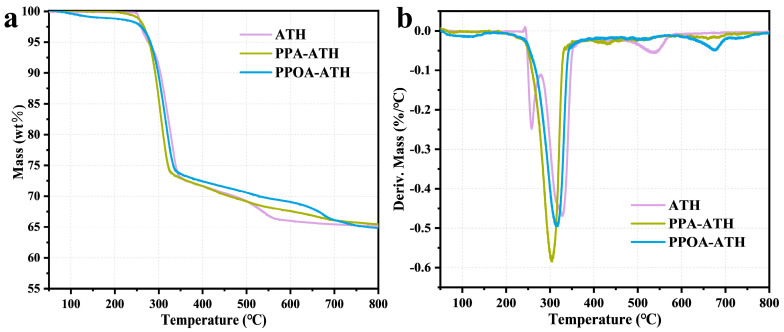
(**a**) TGA and (**b**) DTG curves of ATH, PPA-ATH, and PPOA-ATH under an N_2_ atmosphere.

TGA analysis was performed to investigate the thermal decomposition behavior of ATH before and after modification. As displayed in Figure 5, the T_5%_ (the temperature at 5% mass loss) of ATH is 277 °C, indicating its onset of thermal decomposition. Furthermore, ATH undergoes two main decomposition stages. The first decomposition process takes place from 243 °C to 278 °C, primarily due to the formation of the AlOOH intermediate accompanied by water vapor release (Equation (1)) [25]. Following this, the second stage occurs from 278 °C to 359 °C, indicating the subsequent transformation to Al_2_O_3_ (Equation (2)) [26]. Following modification, the T_5%_ of PPA-ATH and PPOA-ATH were 278 °C and 281 °C respectively, showing no obvious variation compared to ATH. However, both samples demonstrated a slight weight loss below 250 °C, attributed to the decomposition of grafted flame retardant components. As the temperature increased, the phosphoric acid generated by PPA-ATH and PPOA-ATH catalyzed the chemical reaction of ATH and promoted char residue formation [27]. Moreover, PPOA-ATH displayed an additional decomposition peak at approximately 663 °C, likely due to the decomposition of less stable char residues.

Al(OH)_3_ (s) → AlOOH (s) + H_2_O (g) (1)

2AlOOH (s) → Al_2_O_3_ (s) + H_2_O (g) (2)

The S_BET_ results are summarized in Table 1 to demonstrate the change in the specific surface area of ATH from ball milling modification. The raw ATH exhibited a relatively low S_BET_ of 0.7715 m^2^/g. Following ball milling, a substantial increase in the specific surface area was observed, with PPA-ATH and PPOA-ATH reaching 6.3614 m^2^/g and 12.0903 m^2^/g, respectively. This significant enhancement in the S_BET_ can be attributed to the high-energy ball milling effect [28]. Overall, all of the above characterization results confirm the successful modification of ATH via ball milling.

### 3.2. Dispersion of ATH in EVA Matrix and Tensile Properties of EVA Composites

**Figure 6:**
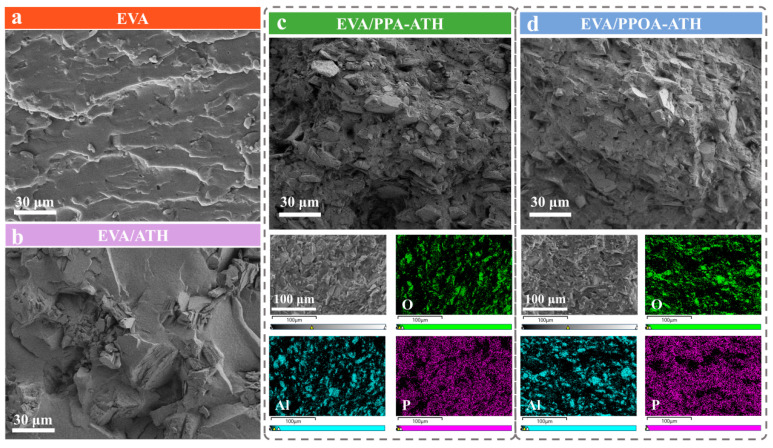
SEM images of freeze-fractured surfaces of (**a**) EVA, (**b**) EVA/ATH, (**c**) EVA/PPA-ATH, and (**d**) EVA/PPOA-ATH composites. Freeze-fractured surface EDS and elemental mapping images of (**c**) EVA/PPA-ATH and (**d**) EVA/PPOA-ATH composites.

The dispersibility of the flame retardant within the composite matrix plays a critical role in affecting its flame retardancy and mechanical properties [29]. Figure 6a,b present SEM images of the freeze-fractured surface of EVA and EVA/ATH. ATH tends to agglomerate, resulting in poor dispersibility within the matrix. In contrast, PPA-ATH and PPOA-ATH demonstrated a more uniform dispersion. EDS images provided a more detailed view of the filler dispersion within the EVA matrix. As displayed in Figure 6c,d, elements O, Al, and P were evenly distributed within the EVA matrix. The improved compatibility is due to their smaller particle size after ball milling, which allowed for easier distribution within the composites. In addition, the hydrogen bonds formed between the PPA/PPOA and the matrix enhanced the interactions, further promoting dispersibility [30].

**Figure 7:**
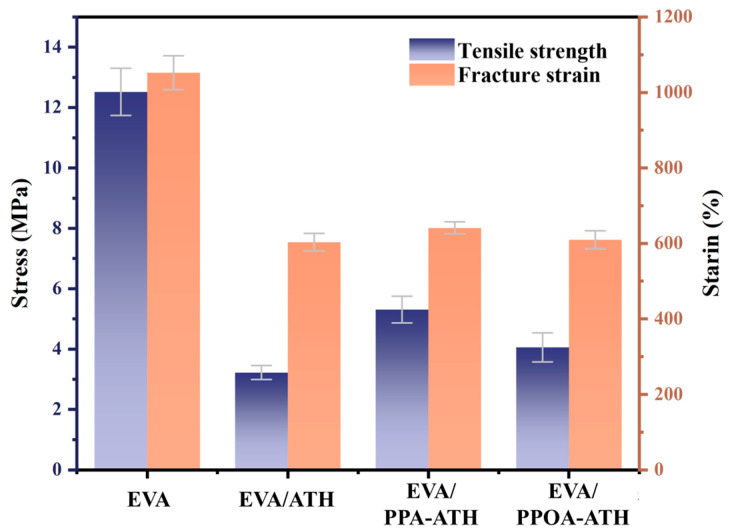
Tensile property results of EVA and its composites.

The tensile properties of EVA and its composites are displayed in Figure 7. Pure EVA exhibits satisfactory tensile properties, with a tensile strength of 12.5 MPa and an elongation at break of 1051.1%. The incorporation of flame retardant inevitably reduced the tensile properties of the composites. Specifically, there was a 74.4% decrease in the tensile strength (from 12.5 to 3.1 MPa) and a 43.1% decrease in the elongation at break (from 1051.1 to 598.4%) for EVA/ATH. Notably, the ball milling modification mitigated the decline trend caused by ATH. As for EVA/PPA-ATH, compared to EVA/ATH, the tensile strength and elongation at break increased by 69.3% (from 3.1 to 5.3 MPa) and 7.1% (from 598.4 to 640.7%), respectively. Moreover, PPOA modification led to a 32.3% increase in the tensile strength (from 3.1 to 4.1 MPa) and a 1.5% enhance in the elongation at break (from 598.4 to 607.2%). These improvements confirm that improved compatibility means better tensile mechanical properties.

### 3.3. Thermal Stability of EVA and Its Composites

**Figure 8:**
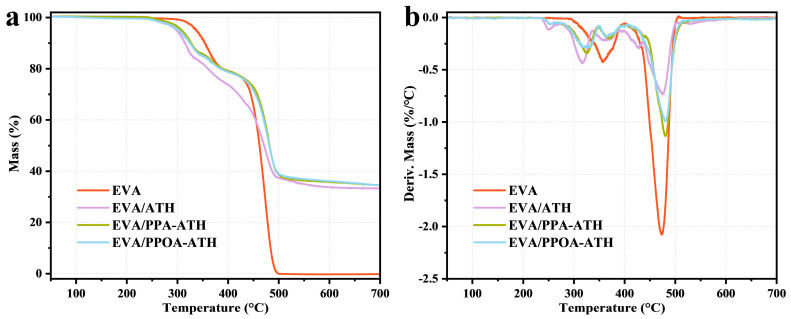
(**a**) TGA and (**b**) DTG curves of EVA and its composites under an N_2_ atmosphere.

To investigate the impact of ATH, PPA-ATH, and PPOA-ATH on the thermal stability of EVA composites, TGA was conducted, and its results are presented in Figure 8 and Table 2, including the T_5%,_ the temperature at 50% mass loss (T_50%_), the maximum thermal degradation temperature (T_max_), and the char yield at 700 °C. Neat EVA undergoes two thermal decomposition stages: the first stage (294–401 °C) corresponds to the loss of acetic acid, while the second stage (401–505 °C) presents the breaking of the main chain [26]. Notably, pure EVA exhibited negligible char-formation ability at elevated temperatures, with no char residues at 700 °C. The incorporation of ATH slightly reduced the onset decomposition temperature from 337 °C for EVA to 299 °C for EVA/ATH due to the early decomposition around 251 °C (consistent with ATH’s thermal behavior). Despite this, the Al_2_O_3_ barrier generated by ATH effectively delayed the second T_max_, reduced the mass loss rate notably, and enhanced the char-forming ability of the composites, resulting in a char residue of 33.3% and demonstrating the positive impact of ATH on thermal stability. As for EVA/PPA-ATH and EVA/PPOA-ATH, their TGA curves exhibit high similarity. The presence of PPA and PPOA promoted the generation of phosphoric acid, which aided in early-stage char formation. The char produced by PPA-ATH and PPOA-ATH was more effective in hindering the decomposition of EVA, as evidenced by the lower mass loss rates at about 366 °C and 475 °C. The above results demonstrate the effect of ATH fillers on enhancing the thermal stability of EVA composites.

### 3.4. Flame Retardancy of EVA and Its Composites

The flame retardancy of the EVA composites was initially evaluated using UL-94 vertical burning tests. As shown in Table 3, the neat EVA was susceptible to ignition, resulting in severe dripping without a UL-94 rating. After the addition of ATH, the dripping of the composites was reduced. This improvement is likely due to ATH’s ability to increase melt viscosity, which helps to prevent melt-dripping during combustion [31]. Unfortunately, EVA/ATH still does not have any UL-94 rating. When PPA-ATH was incorporated, the composites kept burning with a small flame after the initial ignition without a UL-94 rating either. In contrast, EVA/PPOA-ATH exhibited a short t_1_ during the UL-94 tests and achieved a rating of V-2 finally. The difference in performance between the PPA-ATH and PPOA-ATH composites can be attributed to the more effective char formation of PPOA-ATH, which provided better protection against flame exposure.

**Figure 9:**
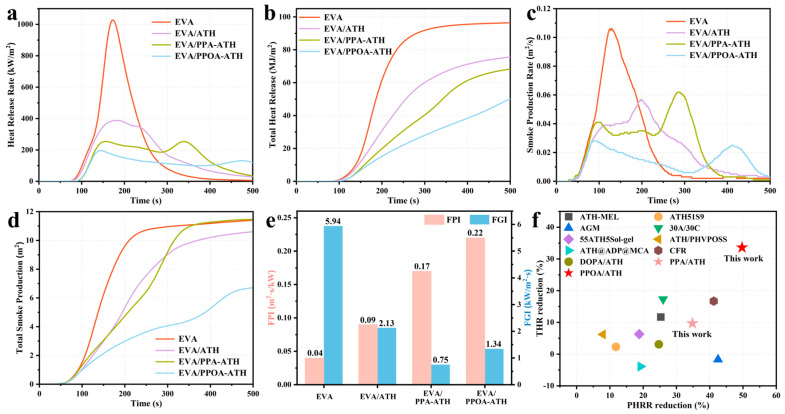
(**a**) HRR, (**b**) THR, (**c**) SPR, (**d**) TSP curves, and (**e**) FPI and FGI results of EVA and its composites. (**f**) Performance comparisons among ATH-based flame retardant systems.

Subsequently, cone calorimetry tests were conducted to further study the combustion behavior of EVA and its composites. The results and key dates are summarized in Figure 9 and Table 4, including the time to ignition (TTI), heat release rate (HRR), peak HRR (PHRR), time to PHRR (t-PHRR), total heat release (THR), fire performance index (FPI), fire growth index (FGI), smoke production rate (SPR), peak PSR (PSPR), and total smoke production (TSP). Neat EVA demonstrated high flammability, igniting in 36 s and releasing substantial heat, with a PHRR of 1027 kW/m^2^ and a THR of 96.4 MJ/m^2^. The addition of inorganic flame retardant ATH significantly reduced the heat release of the composites, resulting in a 62.2% reduction in the PHRR (from 1027 to 388 kW/m^2^) and a 21.7% reduction in the THR (from 96.4 to 75.5 MJ/m^2^), indicating its prior heat-reducing effectiveness. Moreover, the ball milling modification enhanced the flame retardancy of ATH even further. Specifically, the TTI for the PPA-ATH and PPOA-ATH composites was delayed (from 36 to 43 and 42 s, respectively), which can be attributed to the uniform dispersion of flame retardants within the matrix, effectively hindering the transfer of heat and combustion gases before ignition. Additionally, compared with raw EVA, EVA/PPA-ATH led to a 75.4% reduction in the PHRR (from 1027 to 253 kW/m^2^) and a 29.3% reduction in the THR (from 96.4 to 68.2 MJ/m^2^), while EVA/PPOA-ATH exhibited a better flame retardancy, with an 81.0% reduction in the PHRR (from 1027 to 195 kW/m^2^) and a 48.1% reduction in the THR (from 96.4 to 50.1 MJ/m^2^). These improvements can be attributed to the catalyzing effect of PPA-ATH and PPOA-ATH on char formation, as evidenced by the appearance of an earlier HRR peak (which appeared around 140 s) than neat EVA (which appeared at 171 s). The formation of the char layer can effectively prevent heat release and thus protect the composites against flame [32,33]. Furthermore, it is noteworthy that the t-PHRR of EVA/PPA-ATH was 337 s, which is significantly longer than that of the other composites. This result can be attributed to the weakness of its char layer, which failed to protect the composites and was disrupted by heat and smoke during combustion. In addition, it was reported that PPOA can release PO· and PO_2_· free radicals, which can scavenge or quench highly reactive free radicals in the gas phase, further enhancing flame retardancy [34]. Moreover, the FPI and FGI values (where FPI = TTI/PHRR and FGI = PHRR/t-PHRR, with higher FPI and lower FGI values indicating lower fire hazards) follow a similar trend to heat release [35]. The above results demonstrate the superior flame retardancy of PPOA-ATH, which outperformed the ATH and PPA-ATH in forming a more effective protective char layer.

Smoke is a critical parameter in assessing fire hazards [36]. The smoke production of the EVA composites decreased with the addition of flame retardants, with the best smoke-suppression property achieved by EVA/PPOA-ATH. Specifically, EVA/PPOA-ATH reached reductions of 73.3% (from 0.105 to 0.028 m^2^/s) and 41.2% (from 11.4 to 6.7 m^2^) in the PSPR and TSP, respectively. However, it is surprising that PPA-ATH had a poorer smoke suppression ability than ATH, which can also be attributed to its less effective char layer, unable to block heat and smoke adequately.

To demonstrate the flame retardancy enhancement of ball-milling-modified ATH in this work compared to pure ATH, we conducted a comprehensive comparison with ATH-based flame retardant systems and pure ATH in flame-retardant EVA composites reported in the last decade. The related results are represented in Figure 9f and Table 5. Previous research on ATH-based flame-retardant EVA has primarily focused on the physical blending methods, which offer limited flame retardancy enhancement over neat ATH. In this work, PPA-ATH and PPOA-ATH exhibited significant improvements in flame retardancy, especially PPOA-ATH. Specifically, PPOA-ATH achieved remarkable reductions of 49.7%, 33.6%, 50.0%, and 36.8% in the PHRR, THR, PSPR, and smoke production reduction, respectively, compared to the performance of raw ATH in flame-retardant EVA, which can be attributed to the successful introduction of high-efficiency flame-retardant PPOA. These results highlight a substantial enhancement in flame retardant efficiency, underscoring great promise for the fabrication of ATH-based flame retardants.

### 3.5. Char Residues Analysis

**Figure 10:**
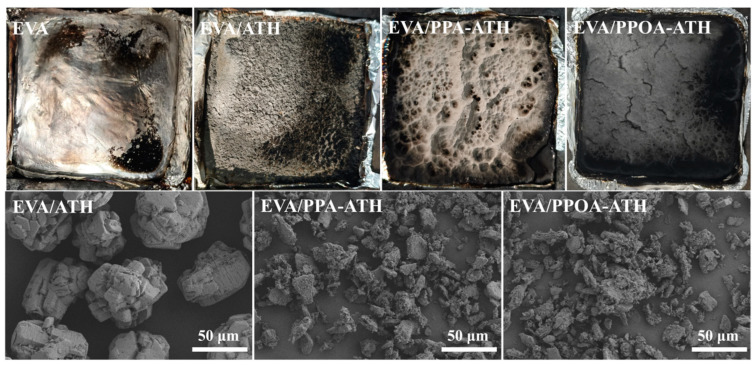
Digital photographs and SEM images of char residues in EVA and its composites.

Digital photographs and SEM images of the char residues after the cone calorimetry test are shown in Figure 10. The neat EVA burned completely with no residue, indicating a low char formation capability. In contrast, a distinct char layer can be observed in the char residue of EVA/ATH. The SEM images of EVA/ATH display the well-dispersed micromorphology of Al_2_O_3_, indicating that ATH generated a char layer composed of Al oxide during combustion and thus protected the matrix. As for EVA/PPA-ATH and EVA/PPOA-ATH, denser char layers were obtained. Their SEM images reveal a more crosslinked char structure compared with that of EVA/ATH, exhibiting their promotional effects on char crosslinking [45].

**Figure 11:**
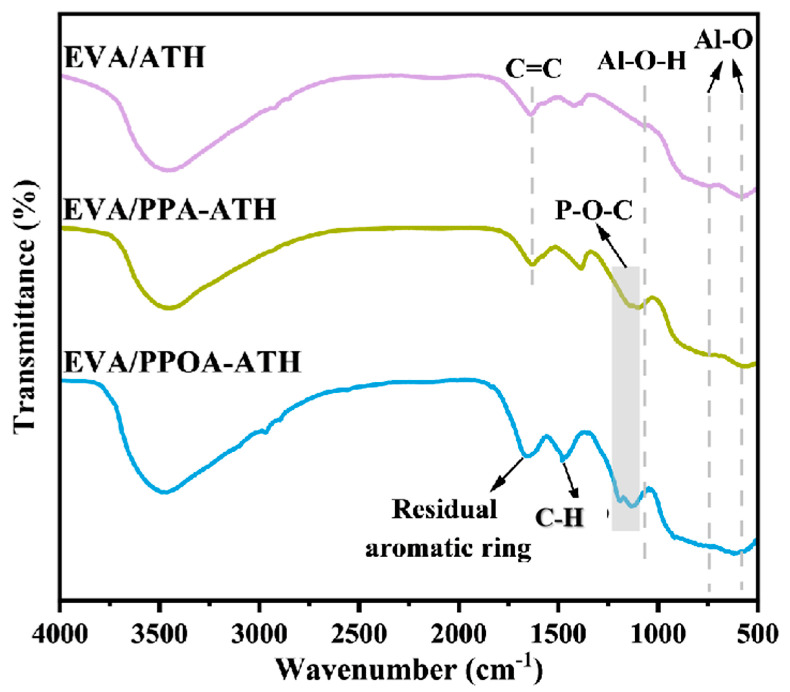
FTIR spectra of the char residues in EVA/ATH, EVA/PPA-ATH, and EVA/PPOA-ATH.

The FTIR spectra are displayed to investigate the composition of the residual chars of EVA/ATH, EVA/PPA-ATH, and EVA/PPOA-ATH. As demonstrated in Figure 11, the spectrum of EVA/ATH exhibits characteristic peaks of Al_2_O_3_ at 1072, 732, and 595 cm^−1^ [46]. Additionally, the absorption peak at 1629 cm^−1^ can be associated with C = C bonds [47]. As for the spectrum of EVA/PPA-ATH, new peaks associated with flame retardant appear. Specifically, the band around 1150 cm^−1^ indicates the presence of P-O-C [48], suggesting that PPA participates in the char formation process. In the case of EVA/PPOA-ATH, the intensity of the P-O-C peak is stronger than that of EVA/PPA-ATH. Furthermore, additional peaks of C-H (1475 cm^−1^) and residual aromatic ring structure (1630 cm^−1^) can be observed [49]. These results indicate that a more stable char layer formed with the addition of PPOA-ATH, explaining the differences in flame retardancy between PPA-ATH and PPOA-ATH.

### 3.6. Flame Retardant Mechanism

Based on the aforementioned results, this work proposes a flame retardant mechanism of EVA/PPA-ATH and EVA/PPOA-ATH composites. During the heating process, the well-dispersed PPA-ATH and PPOA-ATH decompose and absorb heat by generating vapor, which delays the composites’ TTI. During combustion, PPA and PPOA catalyze char formation with ATH, leading to the creation of a cross-linked char layer composed of Al_2_O_3_, which effectively reduces the release of heat and smoke. Additionally, the addition of modified ATH increases the melt viscosity, helping to prevent melt dripping. In the case of the gas phase, the flame retardants lead to the release of P-containing fragments (PO_2_ and PO), inhibiting the decomposition of the composites. Consequently, the fire safety of the composites is significantly improved due to the flame retardant effects of PPA and PPOA in both the condensed and gas phases. Among them, PPOA is more favored in the formation of a more cross-linking protective char layer compared to PPA, resulting in greater heat and smoke reduction and thus achieving the best flame retardancy.

## 4. Conclusions

In this work, high-performance ATH-based flame retardants PPA-ATH and PPOA-ATH were successfully fabricated using a feasible and environmentally friendly ball milling modification method without organic solvent pollution. The modification strategy promoted the grafting of highly flame retardant elements, a reduction in particle size, and an enhancement in the S_BET_, contributing to the dispersion uniformity within the EVA matrix and enhanced thermal stability and flame retardancy of the composites. Among them, PPOA-ATH demonstrated exceptional flame retardant performance, remarkably outperforming pure ATH. The incorporation of PPOA-ATH effectively reduced the heat and smoke release from the composites, with significant reductions of 81.0% in the PHHR, 48.1% in the THR, 73.7% in the PSPR, and 41.2% in the TSP compared to neat EVA composites. These improvements can be attributed to the superior char formation capability of PPOA-ATH, generating a crosslinked protective char layer to reduce heat and smoke release. Furthermore, comparative analysis revealed that the flame retardancy enhancement of PPOA-ATH surpassed most previously reported ATH-based systems. This work provides a feasible, high-efficiency, and environmentally friendly processing ball milling method for the rapid surface modification of ATH, offering a promising pathway for developing high-performance ATH-based flame retardant requirements.

## Figures and Tables

**Table 1:** S_BET_ of ATH, PPA-ATH, and PPOA-ATH.

Parameter	ATH	PPA-ATH	PPOA-ATH
**S_BET_ (m^2^/g)**	0.7715	6.3614	12.0903

**Table 2:** TGA data of EVA and its composites under an N_2_ atmosphere.

Condition	Sample	T_5%_ (°C)	T_50%_ (°C)	T_max1_ (°C)	T_max2_ (°C)	Char Yield at 700 °C (wt%)
N_2_	**EVA**	337	461	356	473	0
	**EVA/ATH**	299	471	312	475	33.3
	**EVA/PPA-ATH**	308	481	323	481	34.6
	**EVA/PPOA-ATH**	303	482	324	480	34.5

**Table 3:** UL-94 vertical combustion results of EVA and its composites.

Sample	UL-94 Vertical Combustion				
	t_1_ * (s)	t_2_ * (s)	Dripping	Cotton Ignited	Rating
**EVA**	>30	-	Heavy	Yes	NR
**EVA/ATH**	>30	-	Slightly	Yes	NR
**EVA/PPA-ATH**	>30	-	Slightly	Yes	NR
**EVA/PPOA-ATH**	<1	12	Slightly	Yes	V-2

* Flame duration after the first and second ignition.

**Table 4:** Cone calorimetry test data of EVA and its composites.

Parameters	EVA	EVA/ATH	EVA/PPA-ATH	EVA/PPOA-ATH
**TTI (s)**	36	35	43	42
**PHRR (kW/m^2^)**	1027	388	253	195
**t-PHRR (s)**	173	182	337	145
**THR (MJ/m^2^)**	96.4	75.5	68.2	50.1
**FPI (m^2^·s/kW)**	0.04	0.09	0.17	0.22
**FGI (kW/m^2^·s)**	5.94	2.13	0.75	1.34
**PSPR (m^2^/s)**	0.105	0.056	0.062	0.028
**TSP (m^2^)**	11.4	10.6	11.4	6.7

**Table 5:** The comparison of the enhancement in flame-retardant EVA composites by ATH-based systems versus pure ATH.

Flame Retardant Systems	Flame Retardant Loading (wt%)	PHRR Reduction (%)	THR Reduction (%)	PSPR Reduction (%)	Total Smoke Reduction (%)	Ref.
**ATH-MEL**	56	25.3	11.7	Not given	Not given	[37]
**ATH51S9**	60	11.8	2.3	Not given	Not given	[38]
**AGM**	40	42.5	−1.7	Not given	Not given	[39]
**30A/30C**	60	26	17.3	Not given	68.1	[40]
**55ATH5Sol-gel**	60	18.8	6.3	Not given	62.6	[41]
**ATH/PHVPOSS**	60	7.9	6.2	57.4	40.9	[42]
**ATH@ADP@MCA**	55	19.2	−3.9	12.3	−54.4	[26]
**CFR**	55	41.2	16.7	53.1	67.9	[43]
**DOPA/ATH**	50	24.7	3.1	Not given	35.4	[44]
**PPA-ATH**	50	34.8	9.7	−10.7	−7.5	This work
**PPOA-ATH**	50	49.7	33.6	50.0	36.8	

**Abbreviations: MEL**: melamine; **S**: silica content; **AGM**: ATH/graphene nanoplatelets/molybdenum disulfide mixtures; **A**: ATH; **C**: calcium borate; **PHVPOSS**: phenyl/vinyl polysilsesquioxane; **ATH@ADP@MCA**: core–shell aluminum hydroxide flame retardant; **CFR**: AlPO_4_@Al(OH)_3_; **DOPA**: dibenzo[c,e][1,2]oxaphosphinic acid.

## Data Availability

The original contributions presented in the study are included in the article, further inquiries can be directed to the corresponding author.
